# Virulence Inhibitors from Brazilian Peppertree Block Quorum Sensing and Abate Dermonecrosis in Skin Infection Models

**DOI:** 10.1038/srep42275

**Published:** 2017-02-10

**Authors:** Amelia Muhs, James T. Lyles, Corey P. Parlet, Kate Nelson, Jeffery S. Kavanaugh, Alexander R. Horswill, Cassandra L. Quave

**Affiliations:** 1 Center for the Study of Human Health, Emory University, 550 Asbury Circle, Candler Library 107E, Atlanta, Georgia, USA; 2 Department of Microbiology, Roy J. and Lucille A. Carver College of Medicine, University of Iowa, Iowa City, IA, USA; 3 Department of Dermatology, Emory University School of Medicine, 615 Michael St., Rm 105L WhiteheadBldg, Atlanta, GA, USA

## Abstract

Widespread antibiotic resistance is on the rise and current therapies are becoming increasingly limited in both scope and efficacy. Methicillin-resistant *Staphylococcus aureus* (MRSA) represents a major contributor to this trend. Quorum sensing controlled virulence factors include secreted toxins responsible for extensive damage to host tissues and evasion of the immune system response; they are major contributors to morbidity and mortality. Investigation of botanical folk medicines for wounds and infections led us to study *Schinus terebinthifolia* (Brazilian Peppertree) as a potential source of virulence inhibitors. Here, we report the inhibitory activity of a flavone rich extract “430D-F5” against all *S. aureus* accessory gene regulator (*agr*) alleles in the absence of growth inhibition. Evidence for this activity is supported by its *agr*-quenching activity (IC_50_ 2–32 μg mL^−1^) in transcriptional reporters, direct protein outputs (α-hemolysin and δ-toxin), and an *in vivo* skin challenge model. Importantly, 430D-F5 was well tolerated by human keratinocytes in cell culture and mouse skin *in vivo*; it also demonstrated significant reduction in dermonecrosis following skin challenge with a virulent strain of MRSA. This study provides an explanation for the anti-infective activity of peppertree remedies and yields insight into the potential utility of non-biocide virulence inhibitors in treating skin infections.

The Brazilian pepper tree or aroeira (*Schinus terebinthifolia* Raddi, Anacardiaceae; also reported under its synonym: *S. terebinthifolius*) is a small dioecious shrub-like tree native to Brazil, Paraguay, and Argentina^1^ (Fig. 1a). A highly invasive species, it is found throughout the southern USA, and Florida, in particular, where it has colonized most of the state and is considered a noxious weed^2^. One of the earliest written records concerning the use of *S. terebinthifolia* date back to 1648 when it was described by Dutch naturalist, Willem Piso, in his book *Historia Naturalis Brasiliae*^3^ (Fig. 1b and Suppl. Table 1). It is included in the Brazilian Pharmacopoeia and has served as a staple in Brazilian traditional medicine^4^ for its anti-septic and anti-inflammatory qualities in the treatment of wounds and ulcers as well as for urinary and respiratory infections^4^,^5^. Bark extracts have demonstrated antibacterial activity against several pathogens, including *S. aureus, Pseudomonas aeruginosa, and Aspergillus* species^6^. Bark extracts were also found to be active against *Pseudomonas aeruginosa*^7^ and were effective against peritonitis when injected into the abdominal cavity of rats^8^.

In addition to anti-infective applications, *S. terebinthifolia* has been used to promote wound healing. When studied in rats with surgically created stomach lesions, treatment with bark extract resulted in accelerated healing^9^. In another study, rats with cutaneous lesions were treated with ointment containing 5% leaf oil, resulting in improved wound healing^10^. Similar findings of improved healing of cutaneous wounds in beef cattle have also been observed following treatment with bark extract for 17 days^11^. Improved clotting and establishment of a fibrin net in the wounds was also observed^11^. A summary of reported antimicrobial bioactivity of this species is provided in Suppl. Table 2 7,12,13,14,15,16. Very little is known, however, regarding the chemistry and bioactivity of the fruits, which were used traditionally as topical poultices for infected wounds and ulcers. Furthermore, while many studies have focused on growth inhibitory, anti-inflammatory, and wound-healing properties of this plant, none have examined its potential as a source of anti-virulence drugs.

Here, we investigate the potential anti-virulence activity of *S. terebinthifolia* against methicillin resistant *Staphylococcus aureus* (MRSA), a pathogen associated with high morbidity and mortality due to its virulence and recalcitrance to antibiotic therapy. MRSA has emerged as a “serious threat” to human health and in 2011, was responsible for 80,461 invasive infections and 11,285 deaths in the USA alone^17^. Though it is a leading cause of healthcare-associated infection, the scourge of MRSA does not remain confined to the hospital setting, but also impacts otherwise healthy individuals in the community. MRSA is able to extend its reach due to the hypervirulent nature of some strains, especially of the USA300 and USA500 lineages. *S. aureus* produces an impressive array of enzymes, hemolysins, and toxins that are essential for its ability to spread through tissues and cause disease^18^. The expression of all of these invasive factors is controlled by quorum-sensing, and the accessory gene regulator (*agr*) system in *S. aureus* mediates this mechanism through the production and sensing of a secreted cyclic peptide signal, also called an autoinducing peptide or AIP^19^,^20^. *S. aureus* has four allelic variants of the *agr* system, each recognizing a unique AIP signal^21^. The *agr* signaling pathway has been thoroughly investigated and detailed studies on its components have been previously reported^20^. Schematic representations of the *agr* system and a description of tools available for detecting small molecules for its inhibition have also been previously reported^22^.

The *agr* locus encodes a peptide based quorum-sensing system that consists of two divergent RNA transcripts, RNAII and RNAIII that are transcribed from the P2 and P3 promoters, respectively. RNAII encodes the machinery for production and sensing of AIP, with the intramembrane protease AgrB processing AgrD to form the mature AIP, and histidine kinase AgrC together with response regulator AgrA comprising a classic two-component system that senses extracellular AIP and upregulates transcription from the P2 and P3 promoters once a critical extracellular AIP concentration is reached. Phosphorylated AgrA also binds to promoters for the loci encoding the alpha and beta phenol soluble modulins (PSM’s), small amphipathic peptides with cytolytic activity, and upregulates their transcription. Increased transcription of RNAIII modulates expression of numerous virulence factors, including cytolytic toxins such as α-hemolysin, since RNAIII functions as a regulatory RNA that controls translation of virulence factor RNA transcripts. In addition, RNAIII encodes a small amphipathic peptide, δ-toxin, that like the PSM’s, possesses cytolytic activity^19^,^20^.

Importantly, quorum-quenching has been demonstrated as an effective strategy to prevent and treat *S. aureus* skin infections^23^,^24^,^25^,^26^. A number of promising candidate molecules have been discovered, including the AgrA inhibitor, savirin^25^, AgrC antagonists solonamide A and B^27^, AgrB inhibitor ambuic acid^28^, the polyhydroxyanthraquinones^29^, and oleanene and ursene derivatives^30^. Based on preliminary studies^31^ that demonstrated the ability of *S. terebinthifolia* fruit extracts to reduce the *agr* -controlled expression of virulence factor *δ*-toxin independent of growth effects, we hypothesized that natural products found in these extracts quench the *S. aureus* quorum sensing system and can be used to improve therapeutic response to MRSA infections, especially related to the skin and soft tissues.

## Results

### Extract 430D-F5 quenches quorum sensing and toxin production

**Figure 2:**
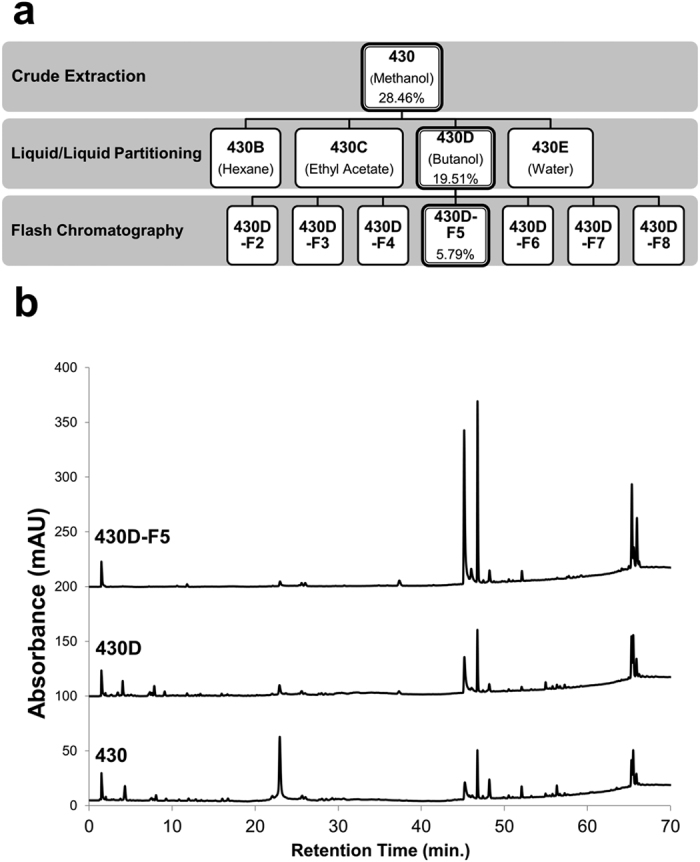
Isolation scheme of bioactive fraction 430D-F5. (**a**) The bioassay-guided fractionation scheme is illustrated, demonstrating the path from crude fruit extract to refined bioactive fraction 430D-F5. Percent yields of extracts in relation to starting dry plant material are represented at each separation step. The most active fractions at each step are highlighted in bold. (**b**) The corresponding HPLC chromatograms for the most active fractions demonstrate an increase in relative abundance of peaks at a retention time range of 45–50 min.

**Figure 3:**
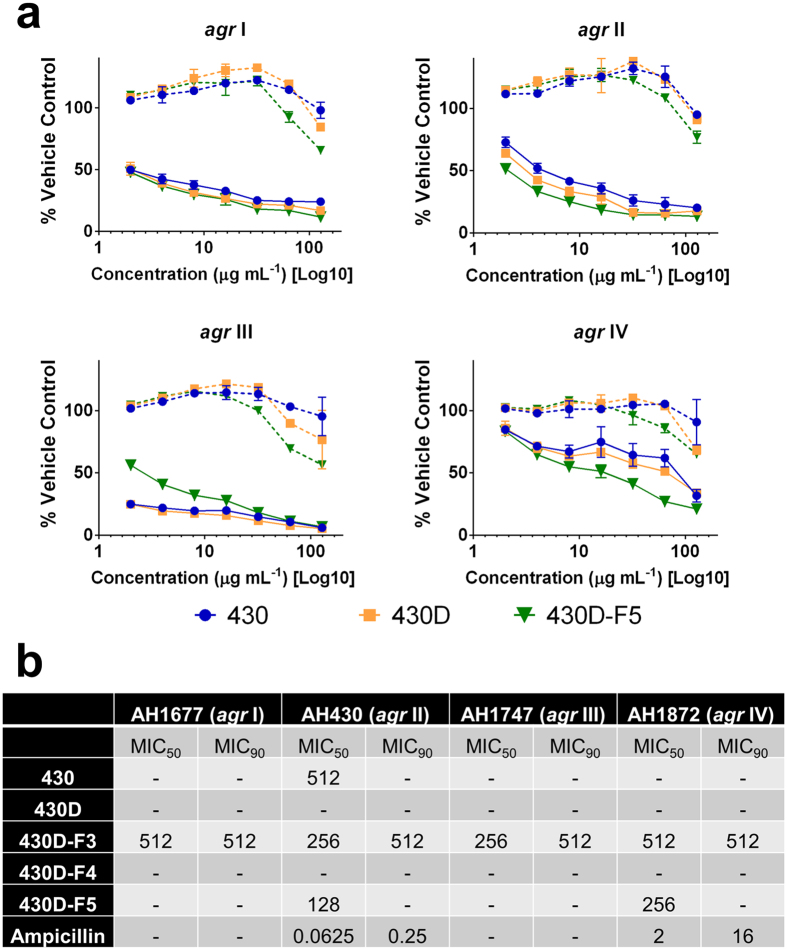
430D-F5 inhibits *agr* activity for all four alleles in a non-biocide manner. (**a**) Data are represented as percent *agr* activity or growth of the vehicle (DMSO) control at 24 hours. The solid lines represent *agr* activity, measured by fluorescence, and the dashed lines represent growth, measured using OD_600_. See Suppl. Table 3 for additional data. (**b**) Minimum inhibitory concentrations (MICs) for 430 and lead fractions, reported as μg mL^−1^.

To refine the most active fraction and potentially identify novel chemical entity inhibitors of *S. aureus* quorum sensing, we employed a bioassay-guided fractionation strategy (Fig. 2) using *agr*-fluorescent reporter stains that represent each of the four known *agr* allelic groups. Strains were grown in the presence of extracts and monitored for growth inhibition by optical density and *agr* activity by fluorescence. Extract 430D-F5 inhibited *agr* at sub-inhibitory concentrations for growth in *agr* I-IV (Fig. 3 and Suppl. Table 3). An upper limit of 512 μg mL^−1^ was used in growth inhibition assays, and the minimum inhibitory concentration for 90% growth inhibition (MIC_90_) value was not detected at this range for 430D-F5, but MIC_50_ values were detected at 128 and 256 μg mL^−1^ for *agr* II and IV, respectively. An upper limit of 128 μg mL^−1^ was used for detecting anti-*agr* activity, and inhibitory concentrations for 50% of the untreated control (IC_50_) values in the *agr* fluorescent reporter assay were 2, 4, 4, and 32 μg mL^−1^ for *agr* I-IV, respectively. An IC_90_ value could only be detected for *agr* III and was found to be 128 μg mL^−1^.

**Figure 4:**
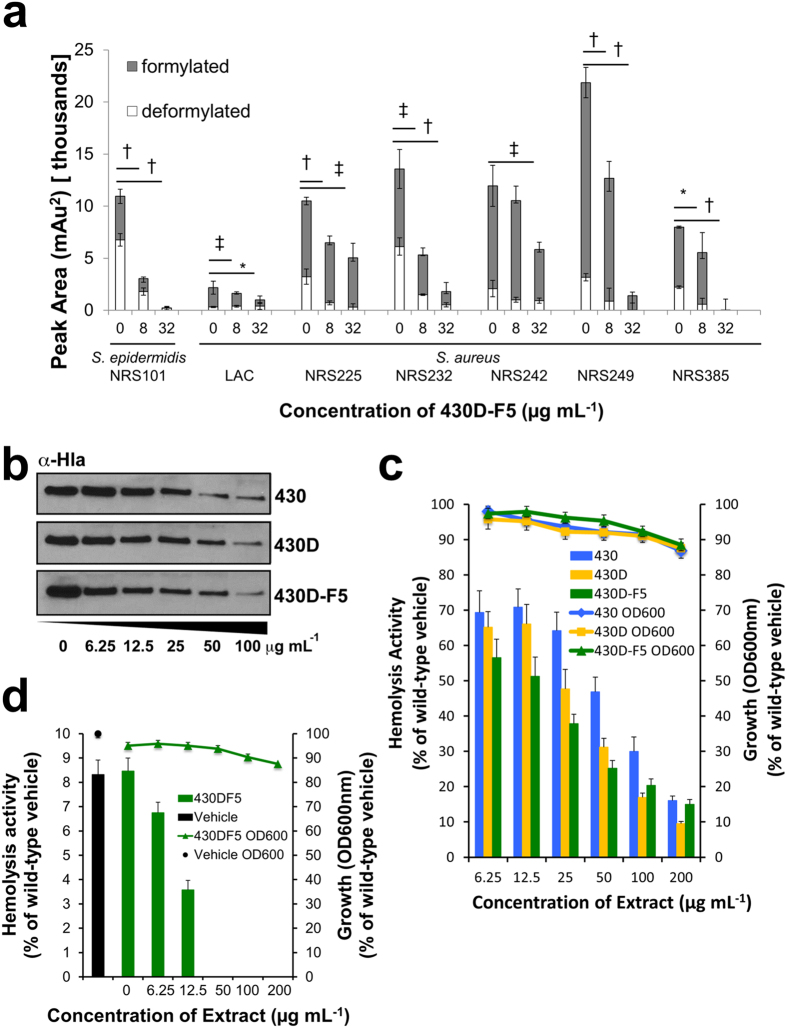
430D-F5 inhibits δ-toxin and α-hemolysin production in a dose-dependent manner. (**a**) Levels of δ-toxin were quantified by HPLC analysis of culture supernatant following treatment with sub-MIC_50_ concentrations of 430D-F5. *S. epidermidis* strain is NRS101, all others are *S. aureus.* Refer to Suppl. Table 5 for full strain details. Results are expressed as the peak area, normalized for optical density (600 nm) at the time of supernatant harvest. Statistical significance is denoted as *P-value < 0.05, ^‡^*P* < 0.01, ^†^*P* < 0.001. (**b**) Western blot analysis displays dose dependent inhibition of α-hemolysin production in USA300 strain LAC (**c**) Red blood cells exposed to supernatants from 430, 430D and 430D-F5 treated cultures demonstrate dose-dependent inhibition of hemolysis by *S. aureus* (LAC) toxins. All treatments are significant in comparison to wild type vehicle control at *P* < 0.001. (**d**) Dose dependent reduction in hemolysis is also evident in 430D-F5 treated supernatants from a *hla* mutant strain (AH1589). All treatments are significant in comparison to wild type vehicle control at *P* < 0.001. No significant growth inhibition in comparison to the vehicle control was observed.

To further confirm the quorum-quenching effects of 430D-F5, we pursued a number of *in vitro* tests to assess its impact on the translational products of *agr.* Levels of δ-toxin, a PSM responsible for various pathophysiologic effects including cytolysis of red blood cells, neutrophils and triggering inflammatory responses^32^,^33^, were quantified by RP-HPLC of the bacterial supernatant following treatment with 430D-F5 at sub-inhibitory concentrations for growth effects. 430D-F5 was effective in significantly reducing δ-toxin production in *S. epidermidis* and in the six *S. aureus* strains examined (Fig. 4a).

Levels of hemolytic α-hemolysin also were assessed via Western blot and a rabbit red blood cell lysis assay. Co-incubation of the extracts with USA300 strain LAC (AH1263) showed a dose-dependent reduction in α-hemolysin protein (Fig. 4b) and a corresponding decrease in hemolytic activity (Fig. 4c). In addition, incubation of 430D-F5 with a mutant LAC strain in which the α-hemolysin gene had been deleted showed a dose-dependent decrease in hemolytic activity (Fig. 4d) that would be attributable to δ-toxin and the PSMs.

**Figure 5:**
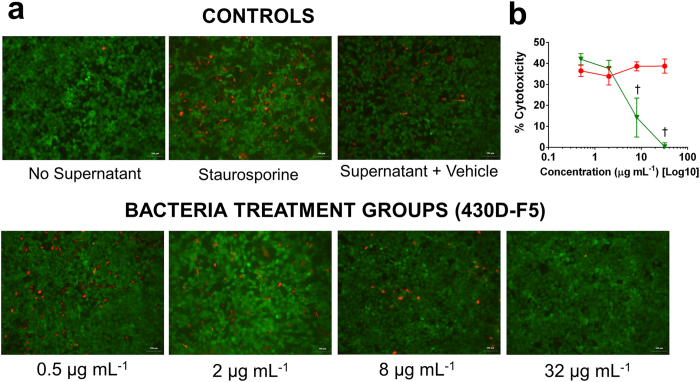
General toxicity of *S. aureus* supernatants to HaCaTs. An immortalized line of human keratinocytes was treated with supernatants of *S. aureus* (NRS385) that were grown +/− 430D-F5 or vehicle (DMSO). Controls with either no supernatant added or staurosporine added were also examined. Both the (**a**) fluorescent microscopy (200X) and (**b**) LDH assay demonstrated that treated cultures lacked the suite of exotoxins in their supernatants, and thus did not impact HaCaT viability.

Lastly, to assess any remaining virulence factors linked to cytotoxicity (e.g., PSMs), sterile filtered supernatants of NRS385 cultures (a virulent USA500 isolate) grown with 430D-F5 were exposed to an immortalized line of human keratinocytes (HaCaTs) and assessed for damage by lactate dehydrogenase (LDH) assay and fluorescent microscopy. HaCaTs were protected from damage by supernatants treated at concentrations as low as 8 μg mL^−1^, and this was confirmed by fluorescent microscopy (Fig. 5a) and LDH assay (Fig. 5b).

### 430D-F5 shows limited potential for impact on skin microbiome

To determine its potential for disrupting the skin microbiome and possibly causing dysbiosis, 430D-F5 was examined for growth inhibitory effects against a panel of nine common skin commensals (Table 1). With the exception of *Propionibacterium acnes* (MIC_50_ of 16 μg mL^−1^), commensal growth was unaffected at the concentration range necessary for *agr*-inhibition in *S. aureus* (IC_50_ of 2–32 μg mL^−1^), suggesting that bactericidal disruption of the skin microbiome would be unlikely. The impact of 430D-F5 on quorum sensing by skin commensals is unknown, and future studies are necessary to determine the impact, if any, on commensal signaling behavior.

### 430D-F5 impacts biofilm formation

**Figure 6:**
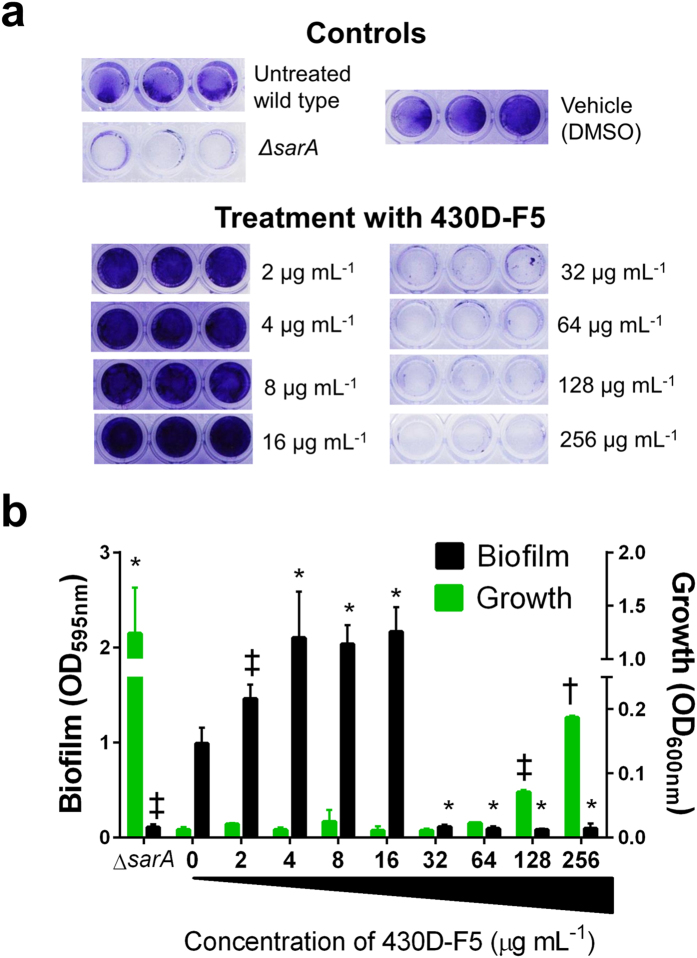
Impact of 430D-F5 on *S. aureus* biofilm formation and planktonic growth in a biofilm model. USA 200 isolate UAMS-1 and its isogenic *sarA* mutant (UAMS-929) were used in the biofilm assay. (**a**) Images of crystal violet stained biofilm in 96-well plates. (**b**) The optical density (OD_595nm_) of the crystal violet eluent is plotted with the OD_600nm_ for planktonic cells, measured by transfer of the well supernatants to a new 96-well plate. Statistical significance in comparison to the vehicle treated wild type control is denoted as *P-value < 0.05, ^‡^*P* < 0.01, ^†^*P* < 0.001.

In microtiter plate biofilm assays, 430D-F5 caused significant, dose-dependent increases in biofilm biomass at concentrations ranging from 2–16 μg mL^−1^, which is consistent with inhibition of *agr* quorum-sensing. In contrast, biofilm biomass was greatly reduced at concentrations of 32 μg mL^−1^ and above, and a dose-dependent increase planktonic cells was observed at the highest concentrations tested (128–256 μg mL^−1^) (Fig. 6), suggesting that at higher dosages 430D-F5 may target more than the *agr* system.

### 430D-F5 is well tolerated by human cells and mouse skin

**Figure 7:**
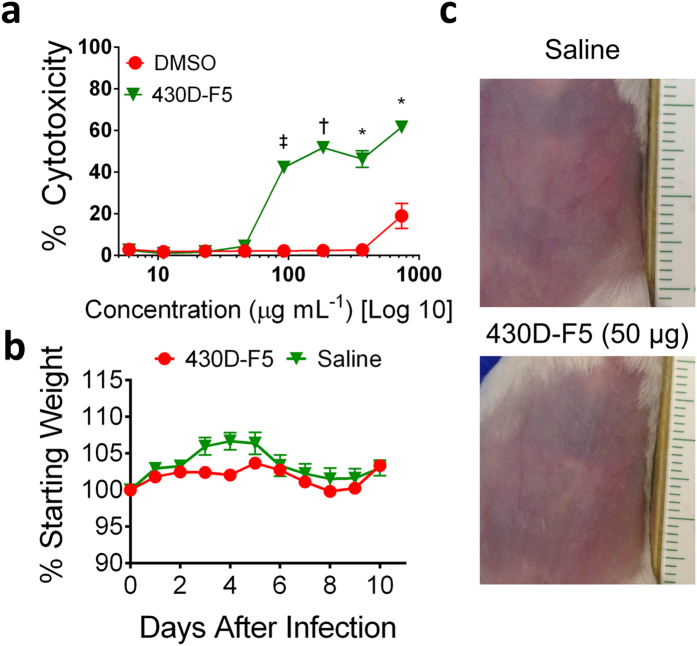
Extracts are non-toxic to human cells and mouse skin at the concentration required for quorum quenching activity. (**a**) Extracts were tested for cytotoxicity using a LDH assay against HaCaT cells at a concentration range of 6–734 μg mL^−1^, with the vehicle (DMSO) not exceeding 1.5% of the well volume. The extracts were non-toxic at concentrations necessary for *agr* inhibition. The IC_50_ values for 430, 430D, and 430D-F5 were 734, 367, and 184 μg mL^−1^, respectively (data not shown). No IC_90_ values were established at this concentration range. (**b**) There was no significant difference in weight gains or losses between mice injected with 50 μg of 430D-F5 or saline. (**c**) No skin irritation or injury were grossly apparent following a 50 μg injection of 430D-F5 into healthy mouse skin (ruler in cm). Photo taken on Day 1.

To determine its potential for toxicity to human cells, the refined fraction (430D-F5) was tested against HaCaTs using an LDH assay for cytotoxicity (Fig. 7a). The IC_50_ was determined to be 128 μg mL^−1^, which yields an initial therapeutic index (TI) of 64 for *agr* I, 32 for *agr* II and III, and 4 for *agr* IV. Furthermore, when administered intradermally to mouse skin, no skin effects (Fig. 7b), morbidity or mortality were noted (Fig. 7c).

### 430D-F5 abates quorum sensing and dermonecrosis *in vivo*

**Figure 8:**
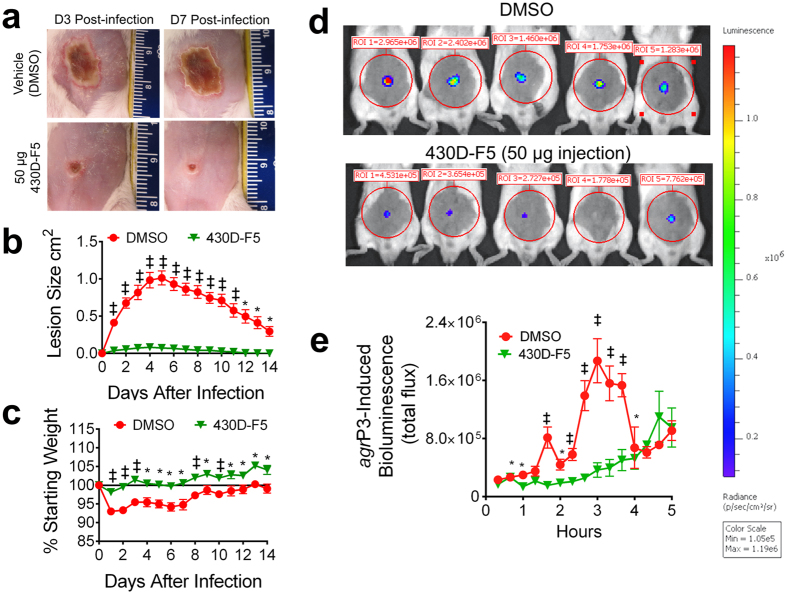
430D-F5 mediates quorum quenching *in vivo* and attenuates MRSA-induced dermatopathology in a murine model of skin and soft tissue infection. (**a**) BALB/c mice were intradermally challenged with an inoculum mixture containing 1 × 10^8^ CFUs of MRSA (LAC) along with either 50 μg of 430D-F5 or the vehicle control (DMSO). Representative images of the resulting cutaneous injuries sustained in 430D-F5 and vehicle control mice are shown for days 3 and 7 post-infection (scale in cm). (**b**) A single 50 μg dose of 430D-F5 profoundly attenuates dermatopathology following cutaneous MRSA challenge. (**c**) 430D-F5 reduces morbidity as measured by animal weight. (**d**) To determine if 430D-F5 inhibits quorum sensing *in vivo*, mice were challenged intradermally with an *agr* P3-lux reporter strain (AH2759) +/− 430D-F5 and *agr-*driven bioluminescence was measured at the indicated time points via IVIS imaging. Quorum sensing peaks at 3 hr post injection, and a single injection of 430D-F5 exhibits significant inhibition of the system for the first 4 h post-injection. (**e**) *In vivo* monitoring demonstrates that 430D-F5 quenches quorum sensing signaling. This image was taken at 3 hr post challenge, during the peak period for *agr* activity. Significant differences between treatment and vehicle are represented as: *P < 0.05; ^‡^*P* < 0.01; ^†^*P* < 0.001.

The potent inhibitory activity of 430D-F5 upon *agr* reporter activity *in vitro* (Fig. 3) encouraged us to assess the efficacy of the mixture within the context of a MRSA infection *in vivo*. Using a previously established mouse model of MRSA^30^ skin challenge, a single dose of 430D-F5 delivered at the time of infection was found to attenuate skin ulcer formation (Fig. 8a and b). In addition, 430D-F5 treated animals exhibited significantly less infection-induced morbidity, as assessed by weight loss, compared to vehicle treated controls (Fig. 8c).

While 430D-F5 clearly attenuated MRSA-induced disease, whether quorum-sensing was inhibited during the course of infection was unclear. To address this question, a MRSA *agr* P3-lux reporter was employed to track *agr* function in real time. Following infectious challenge, we found that the attenuation of MRSA virulence in 430D-F5 treated animals occurred alongside strong and significant suppression of *agr* activity *in vivo* (Fig. 8d and e). Together these data demonstrate that the 430D-F5 mediated attenuation of MRSA pathogenesis corresponds with potent quorum quenching activity both *in vitro* and *in vivo*.

### Chemical characterization of 430D-F5

**Figure 9:**
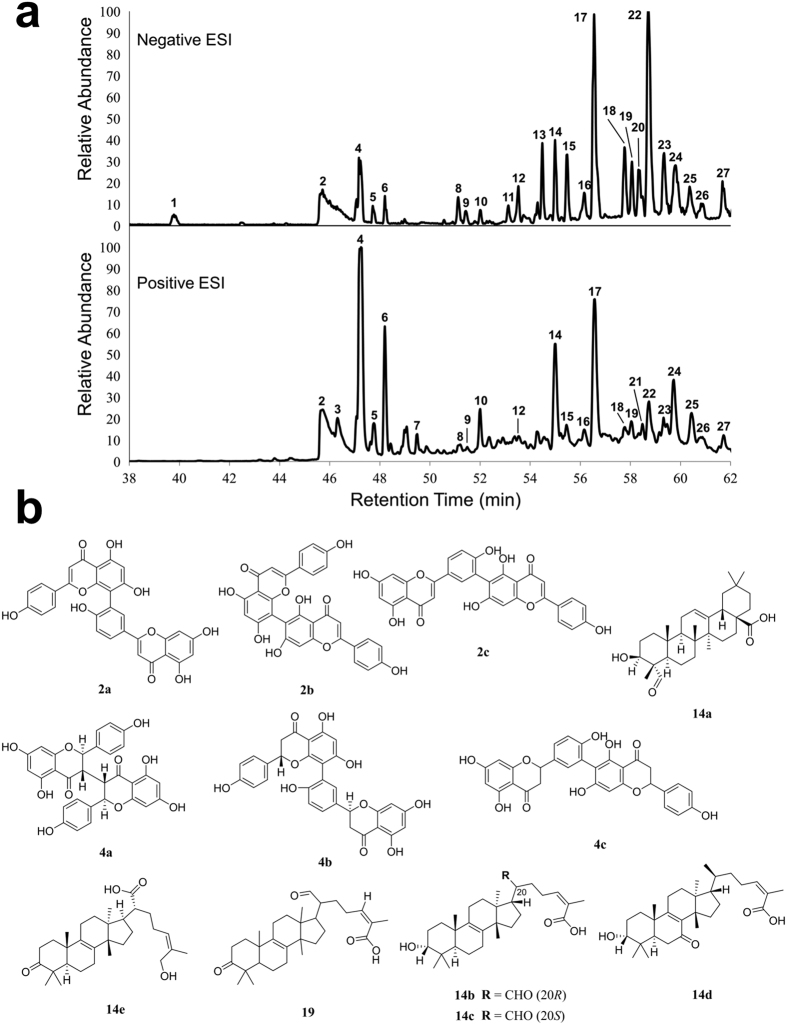
Characterization of 430D-F5 major constituents. (**a**) LC-FTMS ESI negative and positive base peak chromatograms for 430D-F5. All peaks correspond to data presented in Suppl. Table 4. (**b**) Putative structural matches are listed by peak number. Peak **2** was determined to be C_30_H_17_O_10_ and putative structural matches include: (**2a**) amentoflavone, (**2b**) agathisflavone, and (**2c**) robustaflavone. Peak **4** was determined to be C_30_H_21_O_10_ and putative structural matches include: (**4a**) chamaejasmin, (**4b**) tetrahydroamentoflavone, and (**4c**) tetrahydrorobustaflavone. Peak **14** was determined to be C_30_H_45_O_4_ and putative structural matches include: (**14a**) albsapogenin, (**14b**) (13α,14β,17α,20 R,24Z)-3α-hydroxy-21-oxolanosta-8,24-dien-26-oic acid, (**14c**) (13α,14β,17α,20 S,24Z)-3α-hydroxy-21-oxolanosta-8,24-dien-26-oic acid, (**14d**) (3α,13α,14β,17α,24Z)-3-hydroxy-7-oxo-lanosta-8,24-dien-26-oic acid, and (**14e**) mollinoic acid. Peak **19** was determined to be C_30_H_45_O_4_ and putative structural matches include (**19**) isomasticadienonalic acid.

Liquid chromatography Fourier Transform Mass Spectrometry (LC-FTMS) analysis of 430D-F5 yielded 27 peaks with exact masses reported in Suppl. Table 4. Notably, the fraction is rich in flavones and steroidal sapogenins (Fig. 9), exhibiting a different chemical profile than that recently identified in a similar analysis of the quorum quenching activity of a chestnut leaf extract, which was rich in oleanene and ursene derivatives^30^. Peaks **2** and **4** correspond to flavones, representing nearly 34% of the relative abundance in negative electrospray ionization (ESI) mode. Their higher ionization in negative over postitive ESI mode supports the conclusion that these are highly hydroxylated flavone skeletons. The steroidal sapogenins correspond to peaks **14** and **19**, with 5.3% and 1.0% relative abundance respectively. Their lower abundance could also be due to poor ionization in both postive and negative ESI modes.

## Discussion

The goal of this study was to investigate the anti-infective properties of *S. terebinthifolia,* an exotic noxious weed in the US and yet considered a medicinal plant used in treatment of skin infections and wounds in South America. We identified a suite of bioactive compounds in *S. terebinthifolia* fruits that are able to inhibit the *agr* system in *S. aureus* without killing or inhibiting growth. As the global regulator of virulence in *S. aureus,* the *agr* system is responsible for initial infection of host cells, evasion of the host immune system, quorum sensing, and the destruction of tissues with toxins and other enzymes^18^. Through inhibition of the *agr* system with the use of small molecule inhibitors isolated from a medicinal plant, *S. aureus* virulence could be decreased, thus limiting the severity of disease and increasing efficacy of existing antibiotics.

One main concern and point of debate in the literature concerning the utility and limitations of anti-virulence drugs to treat *S. aureus* infection is the potential for activation of other virulence pathways, such as biofilm formation. Here, we found that while at lower concentrations (2–16 μg mL^−1^) there was a slight increase in biofilm formation, there was also an abrupt drop in attached biofilm observed at 32 μg mL^−1^, exhibiting a phenotype similar to that of the Δ*sarA* control (Fig. 6). Although *agr* inhibition typically leads to an enhancement of *in vitro* biofilm formation^34^, the combination of bioactive agents in 430D-F5 clearly prevent biofilm capacity. This observation suggests that at doses required for quorum quenching activity, biofilm formation will also be inhibited, although the anti-biofilm mechanism is currently unknown.

*In vivo* assessment of the most active fraction (430D-F5) demonstrated the proof of concept that by quenching *S. aureus* quorum sensing, it can also inhibit the severity of necrosis at the infection site. Based on previous work by Wright *et al*., which showed that the extent of *S. aureus-*induced dermatopathology is proportional to the magnitude of *agr* activation during the first four hours of infection, we selected this key time period for rigorous tracking of 430D-F5’s effects upon quorum sensing *in vivo*^35^. In our study, the high level of *agr* interference resulting from a single dose of 430D-F5 corresponded with a marked attenuation of MRSA’s infectious phenotype following cutaneous challenge. In future studies, 430D-F5 and isolated small molecules will be assessed for their utility in treating established infections both alone and concomitantly to antibiotic therapy.

Due to its long-standing use as a traditional medicine, a chemically complex preparation of the refined extract would be eligible for consideration of Food and Drug Administration (FDA) approval via the Botanical Drug Regulatory Pathway^36^. These findings support the translational potential for products of this exotic pest plant, and could provide an alternative avenue for use of this natural resource in place of eradication efforts, which resort to toxic herbicides, including glyphosates. Thus, future research will also address alternative management and use strategies for this species as a wild-crafted medicinal plant and potential botanical ingredient in future anti-virulence therapies.

## Methods

### Collection of plant material

*Schinus terebinthifolia* Raddi, Anacardiaceae leaves, stems, and fruits were collected in bulk from private lands in DeSoto County, Florida in November of 2013 and 2014 after obtaining permission from the land owner. Procedures from the 2003 World Health Organization (WHO) Guidelines for good agricultural and collection practices (GACP) for medicinal plants were followed for the collection and identification of bulk and voucher specimens^37^, specifically excluding any populations that may have prior exposure to herbicides. Vouchers were deposited at the Emory University Herbarium (GEO) (Voucher CQ-400, GEO Accession No. 020063) and were identified using the standard Flora for Florida^38^. Plant leaves, stems, and fruits were separated and manually cleaned of soil and contaminants. Plant material was then dried in a desiccating cabinet at low heat. Once dry, plant material was sealed in paper bags and stored at room temperature until further processing.

### Extraction and separation

Crude methanol extracts of fruits were created by blending a ratio of 1 g dry material: 10 mL MeOH into a slurry in a Waring commercial blender for 5 min, and then sonicating the material for 20 minutes. Following decantation of the extract, plant material was subjected to two more rounds of sonication followed by filtration. Filtered extracts were combined, concentrated at reduced pressure with rotary evaporators (<40 °C), and lyophilized. The dried extract was resuspended in 20% MeOH_(aq)_ at 1 g:33 mL and underwent sequential liquid-liquid partitioning three times each with an equal volume of hexane, EtOAc, and H_2_O saturated n-butanol. The organic partitions were dried over Na_2_SO_4_ and filtered. Each partition was concentrated in vacuo at <40 °C. The hexane partition was dissolved and transferred to a tared scintillation vial and dried under forced air to yield 430B. The remaining partitions were suspended in dH_2_O, shell frozen, lyophilized and the resulting powder stored at −20 °C. The EtOAc partition was labeled 430 C, the n-butanol 430D, and final remaining aqueous partition 430E.

### Fractionation by flash chromatography

Following initial quorum quenching assays, the most active partition, 430D was subjected to fractionation through flash chromatography. Fractionation was performed using a CombiFlash® Rf+ (Teledyne ISCO) flash chromatography system with a RediSep Rf Gold silica column. The dry load column was prepared by binding 430D to Celite 545 at a ratio of 1:4. Flash chromatography was performed using a three solvent system of (A) hexane, (B) DCM, and (C) MeOH. The gradient began with 100% A for 6 column volumes (CV), then went to 100% B over 12 CV and was held for 18.2 CV. The gradient was then changed to 74.5:25.5 B:C over the course of 3.1 CV. These conditions were held for 6.8 CV, following which the gradient changed to 68.8:31.2 B:C over 0.7 CV and was held at these conditions for 7.5 CV. Finally, the gradient was adjusted to 100% C over 2.2 CV and held for 14.6 CV. The chromatography was monitored at 254 and 280 nm, as well as via evaporative light scanning detector (ELSD). Tube volumes were combined to create eight fractions: 430D-F1 (tubes 1–5), 430D-F2 (tubes 6–11), 430D-F3 (tubes 12–16), 430D-F4 (tubes 17–24), 430D-F5 (tube 24), 430D-F6 (tubes 25–28), 430D-F7 (tubes 29–31), 430D-F8 (tubes 32–38).

### HPLC characterization of extracts

Extracts 430, 430D, and 430D-F5 were characterized by high performance liquid chromatography (HPLC). An Agilent Eclipse XDB-C18 4.6 × 250 mm, 5-μm analytical column, with a compatible guard column, was used at 40 °C. Active sub-fractions were dissolved in DMSO (10 mg mL^−1^), filtered at 0.2-microns, and a 10 μL injection was eluted at a flow rate of 1.9 mL min^−1^ using a gradient system consisting of (A) 0.1% formic acid in H_2_O; (B) 0.1% formic acid in acetonitrile (ACN). The mobile phase was 98:2 A:B at time 0 min, 70:30 A:B at 40 min, 2:98 A:B at 63 min, followed by a hold at 2:98 A:B for 5 min, and ending with a column flush at initial conditions for 5 min. The chromatography was monitored at 217, 254, 320, and 500 nm.

### Characterization of 430D-F5 by LC-FTMS

LC-FTMS was performed on bioactive fractions using a Thermo Scientific LTQ-FT Ultra MS equipped with a Shimadzu SIL-ACHT and Dionex 3600SD HPLC pump. For chromatography an Agilent Eclipse XDB-C18 4.6 × 250 mm, 5 μm analytical column, with guard column, was used at room temperature. Samples were prepared as previously described and a 50 μL injection was eluted at a flow rate of 1.9 mL min^−1^ using mobile phases of (A) 0.1% formic acid in H_2_O; (B) 0.1% formic acid in ACN. The linear gradient had initial conditions of 95:5 A:B at 0 min and held until 3 min, 68:32 A:B at 23 min, 40:55 A:B at 40 min, 0:100 A:B at 75 min and held for 9 min before returning to initial conditions for a 9 min flush. Data was acquired in MS^1^ mode scanning from a *m/z* of 150–1500 in negative and positive ESI (electrospray ionization) mode and processed with Thermo Scientific Xcalibur 2.2 SP1.48 (San Jose, CA). The capillary temperature was 275.0 °C, sheath gas of 40, source voltage 5.00 kV, source current 100.0 μA, and the capillary voltage −19.0 V or +32.0 V for negative and positive modes, respectively.

Putative formulas were determined by performing isotope abundance analysis on the high-resolution mass spectral data with Xcaliber software and reporting the best fitting empirical formula. Database searches were performed using the Dictionary of Natural Products (Taylor & Francis Group) and Scifinder (American Chemical Society). The databases were reviewed for compounds identified from the genus *Schinus* with molecular masses corresponding to the LC-FTMS data. Any matches were investigated by comparing the literature and the experimental data; putative compound assignments were made when matches were identified.

### Bacterial strains

Quorum sensing reporter strains of *S. aureus* representing the four *agr* groups included AH1677, AH430, AH1747, and AH1872 (Suppl. Table 5), all previously described^39^. AH1677 is an *agr* I reporter from strain AH845 (CA-MRSA USA 300 LAC), AH430 is an *agr* II reporter from SA502A, AH1747 is an *agr* III reporter from strain CA- MRSA MW2, and AH1872 is an *agr* IV reporter from MN EV. All strains contained plasmid pDB59 that served as an *agr* fluorescence reporter and was maintained through culture in media containing chloramphenicol at a concentration of 10 μg mL^−1^. AH2759, a LAC *agr* P3-lux reporter, was maintained in TSB containing chloramphenicol at a concentration of 10 μg mL^−1^. Chloramphenicol (Sigma-Aldrich) was dissolved in 95% EtOH to a stock concentration of 20** **mg mL^−1^ and stored at −20 °C until being added to the media. Cultures were grown on Tryptic Soy Agar (TSA) supplemented with 10 μg mL^−1^ chloramphenicol. Other *S. aureus* strains, LAC, AH3052, UAMS-1, UAMS-929, NRS225, NRS232, NRS239, NRS242, NRS249, and NRS385, and *S. epidermidis* strains, NRS101 and HM896, were grown in Tryptic Soy Agar (TSA) and Tryptic Soy Broth (TSB). *Staphyloccocus warneri* was grown in TSB and Brain-Heart Infusion (BHI) agar. *Staphylococcus haemolyticus, Streptococcus pyogenes, Streptococcus mitis*, and *Corynebacterium amycolatum* were grown in BHI broth and TSA with 5% sheep blood. *Microccocus luteus* was grown in nutrient agar and broth. *Corynebacterium striatum* was grown in TSB and TSA with 5% sheep blood. *Proprionibacterium acnes* was grown in BHI with 1% dextrose and TSA with 5% sheep blood under static, anaerobic conditions.

### Minimum inhibitory concentration

All partitions and fractions of extract 430 were evaluated for minimum inhibitory concentrations (MICs) against the four *agr* groups, the biofilm test strain (UAMS-1) and NRS385 (for δ-toxin quantification experiments) following the Clinical Laboratory Standards Institute (CLSI) M100-S23 guidelines for microtiter broth dilution testing^40^. Briefly, overnight cultures of *S. aureus* were standardized by optical density (OD) t matching to a 0.5 McFarland standard to reach a final inoculum density in the wells of 5 × 10^5^ CFU mL^−1^ in Cation-adjusted Mueller-Hinton Broth (CAMHB), verified by colony plate counts. Two-fold serial dilutions were performed in 96-well plates and incubated in a humidified chamber at 37 °C for 18 h without shaking. The OD_600_ was taken with a BioTek Cytation 3 multimode plate reader at 0 and 18 h to calculate percent inhibition of growth. The MIC_50_ and MIC_90_ are defined as the minimum concentration necessary to inhibit 50% and 90% growth, respectively. Controls included the vehicle (DMSO) and antibiotic, Ampicillin (Amp) (MP Biomedicals Inc). Extract 430D-F5 was additionally assessed against NRS101, LAC, NRS225, NRS232, NRS239, NRS242, NRS249 (for δ-toxin quantification)^40^, and a suite of skin commensals as previously described^30^.

### *Agr* reporter assay

*S. aureus agr* reporter strains AH1677 (*agr* I), AH430 (*agr* II), AH1747 (*agr* III), and AH1872 (*agr* IV) were grown overnight in TSB supplemented with chloramphenicol at 37 °C while shaking at 200 rpm. Cell density was standardized as described above in TSB, supplemented with 10 μg mL^−1^ chloramphenicol. Extracts were tested at 2-fold serial dilutions (2–256 μg mL^−1^). Black sided, 96-well, clear bottom, tissue-culture treated plates (Costar 3603) with final well volume of 200 μL were used for all *agr* inhibition assays. Plates were incubated in humidified chamber at 37 °C while shaking at 260 rpm. After 24 hours, plates were removed and OD_600_ and fluorescence were measured by plate reader at an excitation of 493 nm and emission of 535 nm.

### Quantification of δ-toxin by HPLC

In effort to confirm that the *agr* inhibition observed in the reporter assay corresponded with an actual decline in exotoxin production, the amount of δ-toxin present in the culture supernatant of treated and untreated samples was quantified using NRS385, a USA500 *agr* I strain of *S. aureus*, which has been used for a similar purpose in previous studies^30^,^41^. Briefly, an overnight culture of NRS385 was standardized to 5 × 10^5^ CFU mL^−1^ in TSB and treated with 4-fold serial dilutions containing DMSO, 430 and 430D at 2–32 μg mL^−1^, and 430D-F5 at 0.5–32 μg mL^−1^. Experiments were conducted in quadruplicate in 14 mL snap-cap tubes with a final volume of 1.5 mL. All extracts were tested at sub-MIC_50_ concentrations (Fig. 3b). Tubes were incubated at a 45° angle at 37 °C while shaking (275 rpm) for 15 hours. After incubation, cultures were placed on ice, an aliquot was taken to determine OD, then centrifuged at 13,000 rcf for 5 min at a temperature of 4 °C. Supernatants were removed and 750 μL of each supernatant was placed in a vial for HPLC quantification of δ-toxin. The remaining volume of supernatant was sterile filtered with a 0.22 μm nylon syringe filter and stored at −20 °C until needed for later treatment of cells in the HaCaT cytotoxicity assay.

Delta-toxin was also quantified after treatment with the refined fraction 430D-F5 at 8 and 32 μg mL^−1^ in the following strains: NRS101, LAC, NRS225, NRS232, NRS239, NRS242 and NRS249. Quantification of δ-toxin was performed by HPLC as previously described^42^. Data was normalized across strains based on optical density of the culture prior to centrifugation and harvest of supernatants.

### Hemolytic activity

Hemolysis assays for alpha-hemolysin activity with red blood cells were performed as previously described^30^.

### Western blots

Western blots for alpha-hemolysin (*Hla*) levels were performed as previously described^30^. Briefly, an overnight culture of *S. aureus* AH3052 *Δspa* was inoculated into 5 mL of TSB at 1:500 and grown at 37 °C with shaking (250 rpm), in the presence of either DMSO or one of the extracts (430, 430D, 430D-F5) at concentrations of 6.25, 12.5, 50 and 100 μg mL^−1^. Following 8 hr of incubation, 600 μL of each culture was filter sterilized using a cellulose acetate SpinX 0.22 μm filter (Corning) and the filter sterilized media was stored at −20 °C. The filtered media was electrophoresed on 13% SDS-PAGE gels and transferred to nitrocellulose membranes (Bio-Rad). Membranes were blocked overnight at 4 °C in TBST (20 mM Tris [pH 7.5], 150 mM NaCl, 0.1% Tween 20) with 5% nonfat dry milk then washed 3 times with TBST. Hla was detected using a polyclonal rabbit anti-Hla antibody at a 1:5000 dilution and a goat anti-rabbit HRP secondary antibody (Jackson ImmunoResearch Laboratories) at a 1:20000 dilution. Blots were incubated at RT for 5 min with Supersignal West Pico Chemiluminescent Substrate (Thermo Scientific) then exposed to film for 5 min.

### Biofilm formation

Extract 430D-F5 was examined for impact on *S. aureus* biofilm formation using a human plasma protein-coated assay as previously described^30^,^42^,^43^. Additionally, to monitor for any growth impact in this assay, the optical density of planktonic cells in the biofilm wells was calculated by transferring the supernatant to a new 96-well plate and reading the OD_600nm_ in a BioTek plate reader. Isolate UAMS-1^44^ was used for the biofilm assay, and its isogenic *sarA* mutant (UAMS-929), which has a biofilm deficient phenotype, served as a positive control.

### HaCaT cytotoxicity

The HaCaT cell line was maintained and cytotoxicity of extracts (430, 430D and 430D-F5) and 430D-F5 treated supernatants were assessed using a LDH cytotoxicity assay as previously described^30^. The Therapeutic Index (TI) was calculated as a ratio of the TD_50_ (Toxic dose for cytotoxicity at IC_50_) and ED_50_ (effective dose for quorum quenching IC_50_): 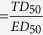. Cytotoxicity of supernatants was further evaluated using a Viability/Cytotoxicity assay and imaged with fluorescent microscopy as previously described^30^.

### Mice and *S. aureus* skin challenge model

A previously described^30^ murine model of MRSA skin infections was used to assess the efficacy of 430D-F5 as an anti-infective. All animal experiments described herein was approved by and conducted in accordance with the recommendations of Animal Care and Use Committee at the University of Iowa (IACUC #1205097). Briefly, LAC was grown in TSB medium overnight at 37 °C in a shaking incubator set to 200 rpm. Overnight cultures were diluted 1:100 in fresh TSB grown (~2 h) to an OD_600 nm_ of 0.5. Bacterial cells were then pelleted and resuspended in sterile saline to a concentration of 1 × 10^8^ CFUs/45 μL. 50 μL inoculum suspensions containing 1 × 10^8^ CFUs and either 430D-F5 (50 μg diluted in DMSO) or DMSO alone were injected intradermally into abdominal skin of age and sex matched BALB/c mice. Baseline body weights of mice were measured before infection and every day thereafter for a period of 14 days. For determination of lesion size, digital photos of skin lesions were and analyzed via ImageJ software.

AH2759, a LAC *agr* P3-lux reporter strain, was used in inoculum preparation for assessing quorum quenching *in vivo*. It was grown in TSB medium containing chloramphenicol at a concentration of 10 μg mL^−1^ overnight at 37 °C in a shaking incubator (200 rpm). Overnight cultures were diluted 1:100 in TSB + chloramphenicol (10 μg mL^−1^) and subcultured to an optical density of 0.1 at 600 nm (≈1 hour). Bacterial cells were then pelleted and resuspended in sterile saline to a concentration of 2 × 10^7^ CFUs/45 μL. Inoculum suspensions (50 μL) containing 2 × 10^7^ CFUs and 50 μg of 430D-F5 diluted in DMSO or DMSO alone were injected intradermally into abdominal skin using 0.3 mL/31 gauge insulin syringe. For all infections, challenge dose was confirmed by plate counts.

### Measurement of quorum quenching *in vivo*

Beginning at 20 minutes post infection, mice were imaged under isoflurane inhalation anesthesia (2%). Photons emitted from luminescent bacteria were collected during a 2 min exposure using the Xenogen IVIS Imaging System and living image software (Xenogen, Alameda, CA). Bioluminescent image data are presented on a pseudocolor scale (blue representing least intense and red representing the most intense signal) overlaid onto a gray-scale photographic image. Using the image analysis tools in living image software, circular analysis windows (of uniform area) were overlaid onto abdominal regions of interest (as depicted in Fig. 8e) and the corresponding bioluminescence values (total flux) were measured and plotted vs. time after infection.

### Statistical analysis

All assays performed were analyzed using a two-tailed Student’s *t*-test with unequal variance as calculated by Microsoft Excel 2010. DMSO treated (vehicle control) cultures were used as a vehicle control and were compared to those treated with extract for all statistical analyses. *P*-values < 0.05 were considered statistically significant. All assays and other experiments were performed in triplicate or quadruplicate.

## Additional Information

**How to cite this article:** Muhs, A. *et al*. Virulence Inhibitors from Brazilian Peppertree Block Quorum Sensing and Abate Dermonecrosis in Skin Infection Models. *Sci. Rep.* **7**, 42275; doi: 10.1038/srep42275 (2017).

**Publisher's note:** Springer Nature remains neutral with regard to jurisdictional claims in published maps and institutional affiliations.

## Supplementary Material

Supplementary Tables

## Figures and Tables

**Figure 1:**
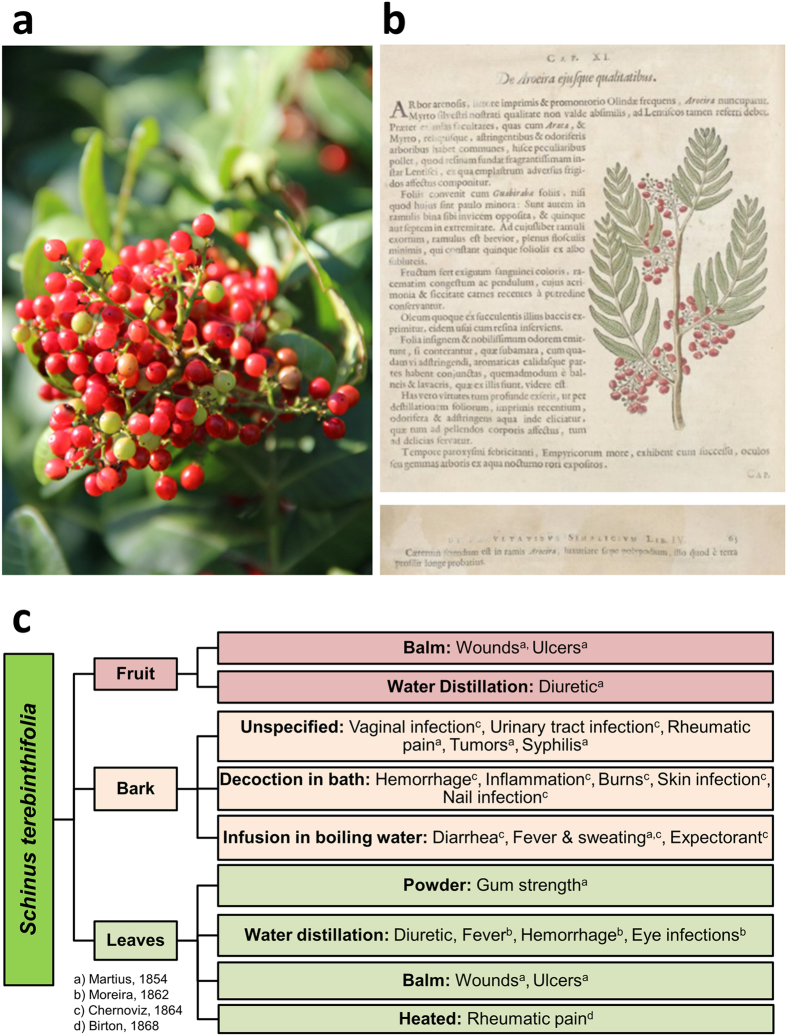
An exotic pest plant to some, a valued source of medicine to others. *Schinus terebinthifolia* Raddi is classified as a Category I pest plant by the Florida Exotic Pest Plant Council. Efforts to remove it from the United States have included the use of the herbicides triclopyr and glyphosate^45^. On the other hand, its value as a medicinal plant has been broadly reported in South America^33^,^46^,^47^. (**a**) *S. terebinthifolia* in fruit (Photo Credit: CL Quave). (**b**) Written historical records of the medicinal uses of *Schinus* sp. plant date back to 1648^3^, appearing in the *Historia Naturalis Brasiliae* by Dutch naturalist, Willem Piso^48^. A full translation of this section of text is provided (Suppl. Table 1). (**c**) Historical traditional medicinal uses and preparations of different tissues from *S. terebinthifolia*^33^,^46^,^47^.

**Table 1:** Antibacterial activity of 430D-F5 on members of the skin microflora.

Species	Strain	MIC	430D-F5	Antibiotic Controls*			
				Amp	Clin	Erm	Van
*Corynebacterium amycolatum*	SK46	MIC_50_	ND (512)	0.0625	—	0.00781	0.5
		MIC_90_	ND (512)	2	—	2	2
*Corynebacterium striatum*	FS-1	MIC_50_	ND (512)	ND (16)	—	1	0.5
		MIC_90_	ND (512)	ND (16)	—	2	0.5
*Micrococcus luteus*	SK58	MIC_50_	64	0.125	0.125	0.0625	0.25
		MIC_90_	128	0.125	0.5	0.0625	0.25
*Propionibacterium acnes*	HL005PA2; HM-493	MIC_50_	16	—	0.125	0.125	—
		MIC_90_	256	—	0.125	0.5	—
*Staphylococcus epidermidis*	NIHLM001; HM896	MIC_50_	64	0.03125	—	—	1
		MIC_90_	ND (512)	0.0625	—	NT	1
*Staphylococcus haemolyticus*	NRS116	MIC_50_	64	ND (32)	—	ND (32)	1
		MIC_90_	ND (512)	ND (32	—	ND (32)	2
*Staphylococcus warneri*	SK66	MIC_50_	64	0.0625	—	—	0.5
		MIC_90_	ND (512)	0.0625	–	—	1
*Streptococcus mitis*	F0392	MIC_50_	64	0.03125	—	0.00781	0.5
		MIC_90_	ND (512)	0.0625	—	0.03125	0.5
*Streptococcus pyogenes*	MGAS15252	MIC_50_	ND (512)	0.0156	0.125	0.0625	—
		MIC_90_	ND (512)	0.0313	0.125	0.0625	—

430D-F5 is nontoxic to several members of the skin microflora at the concentrations required for quorum sensing inhibition in *S. aureus*, but does inhibit growth of *P. acnes.*

^*^Amp: ampicillin; Clin: clindamycin; Erm: erythromycin; Van: vancomycin. —: not tested. ND: MIC not detected (max. concentration tested).
