# Supplementary material for: Virulence Inhibitors from Brazilian Peppertree Block Quorum Sensing and Abate Dermonecrosis in Skin Infection Models
Source: Sci Rep. 2017 Feb 10;7:42275. doi: 10.1038/srep42275 (PMC5301492; doi:10.1038/srep42275)
Supplement: Supplementary Tables [file srep42275-s1.pdf]

Virulence Inhibitors from Brazilian Peppertree Block Quorum Sensing and Abate Dermonecrosis in Skin Infection Models

Amelia Muhs[1*], James T. Lyles[1*], Corey P. Parlet[2*], Kate Nelson[3], Jeffery S. Kavanaugh[2], Alexander R. Horswill[2†], and Cassandra L. Quave[1,3†].

Affiliation

[1]Center for the Study of Human Health, Emory University, 550 Asbury Circle, Candler Library 107E, Atlanta, Georgia, USA

[2*]Department of Dermatology, Emory University School of Medicine, 615 Michael St., Rm 105L Whitehead Bldg., Atlanta, GA, USA

[3]Department of Microbiology, Roy J. and Lucille A. Carver College of Medicine, University of Iowa, Iowa City, IA USA

[*]These authors contributed equally to this work.

[†] Corresponding authors. Correspondence and requests for materials should be addressed to CLQ (email: cquave@emory.edu).

Supplementary Table 1: English translation of *Historia Naturalis Brasiliae* section "*De Aroeria eiusque qualitatibus*". Note the reference to medicinal uses of the plant, including the fruit oils and resin as plasters (topical applications) and aqueous leaf extractions to "drive out disease", most likely taken as an internal medicament. Latin excerpt from: Piso, W. *Historia Naturalis Brasiliae* (Joannes de Laet, 1648).

Original Latin	English Translation
**Cap. XI**<br>**De Aroeira eiusque qualitatibus**<br>Arbor arenosis, littore imprimis & promontorio Olindae frequens, *Aroeira* nuncupatur. Myrto silvestri nostrati qualitate non valde absimilis, ad Lentiscos tamen referri debet. Praeter eximias facultatues, quas cum *Araca*, & Myrto, arboribus habet communes, hisce peculiaribus pollet, quod resinam fundat fragrantissimam instar Lentisci, ex qua emplastrum adversus frigidos affectus componitur.	**Cap. XI**<br>**On Aroeira and its qualities**<br>The tree from sandy places, particularly frequent in shores and promonotories of Olinda [historic city in the state of Pernambuco], is called Aroeira. In its quality not very different from the native wild Myrtle, it must remind one of the Lentisks. Besides certain uncommon properties, which it shares with the Araca & Myrto trees, it flourishes in these in particular, because it pours extremely fragrant resin like that of the Lentisk, out of which one can make a plaster against cold affections.
Foliis convenit cum *Guabirabae* foliis, nisi quod hujus sint Paulo minora: Sunt autem in ramulis bina sibi invicem opposite, & quinque aut septem in extremitate. Ad cujuslibet ramuli exortum, ramulus est brevior, plenus, flosculis minimis, qui constant quinque foliolis ex albo subluteis.	The leaves approximate those of the Guabirabae, except hers are smaller than those of the Paulo: they also are in the branch in pairs opposite each other, & with five or seven ends. They spring up from any branch; the branch is shorter, stout, with minimal flowerettes, which consist of five leaflets of a white-saffron yellow [NT: sub- prefix probably points to a white out of the yellow].
Fructum fert (sert) exiguum sanguinei coloris, racematim congestum ac pendulum, cujus acrimonia & siccitate carnes recentes à putredine conservatur.	It bears fruit of an exiguous blood-color, with clusters of berries dense and hanging down, whose fresh flesh is preserved from rotting by pungency and dryness.
Oleum quoque ex succulentis illius baccis exprimitur, eidem usui cum resina inserviens.	Also, the oil of her succulent berries is pressed out, same use as the useful resin.
Folia insignem & nobilissimum odorem emitunt, si conterantur, quae subamara, cum quadam vi adstringendi, aromaticas calidasque partes habent conjunctas, quemadmodum è balneis & lavacris, quae ex illis siunt, videre est.	The leaves emit a notable & extremely noble odor when ground, slightly bitter, with binding force in a certain place, they have the aromatic and hot parts together, as it can be seen, for example, outside restrooms and baths, which is done out of these.
Has vero virtutes tum profunde exserit, ut per destillationem foliorum, imprimis recentium, odorifera & adstringens aqua inde eliciatur, quae tum ad pellendos corporis affectus, tum ad delicias servatur.	It reveals these true excellent properties so profoundly that for the distillation of the leaves, especially when fresh, a fragrant & binding water may be thence extracted, which at times can drive out disease from the body, at other times can be saved for pleasure.
Tempore paroxysmi febricitanti, Empyricorum more, exhibent cum successu, oculos seu gemmas arboris ex aqua nocturne rori expositos.	In times of feverish paroxysms, they appear with success in the custom of Empyrics, eyes or buds of the tree exposed out of the nocturnal water of the dew.
Caeterum sciendum est in ramis Aroeira, luxuriate saepe polypodium, illo quod è terra prosilit longe probatius.	Another thing that is to be known, the *Aroeira*, often a luxurious polypodium, in its branches that which is out of the ground bursts forth quite far.

Supplementary Table 2: Summary of select previous reports of antimicrobial bioactivity from different tissues of *Schinus terebinthifolia*.

Plant Tissue	Extraction method	Antimicrobial Bioactivity†	Attributed Chemistry*	Ref.
Stem bark	Crude hydroethanolic extract	Anti-viral: Herpes Simplex Virus-1 (EC_{50}: 14 µg mL^{-1}; SI: 22.5)	flavan-3-ols	1
	Aqueous extract	Antifungal: *Trichophyton rubrum* (MIC/MFC: 1 mg mL^{-1}); *Trichophyton. mentagrophytes* (MIC/MFC: 1 mg mL^{-1})	-	2
	Ethanol:water extract	Antifungal: *Trichophyton rubrum* (MIC/MFC: 62.5 µg mL^{-1}); *Trichophyton. mentagrophytes* (MIC/MFC: 62.5 µg mL^{-1})	-	2
	50% Hydroalcoholic extract of	Antibacterial: *Staphylococcus aureus* (MIC: 250 µg mL^{-1}), *Pseudomonas aeruginosa* . (MIC: 1000 µg mL^{-1}), *Bacillus subtilis* (MIC: 1000 µg mL^{-1}) Antifungal: *Candida albicans* . (MIC: 0.49 µg mL^{-1}), *C. parapsilosis* (MIC: 62.5 µg mL^{-1}), *C. tropicalis* . (MIC: 62.5 µg mL^{-1})	-	3
Stems	Dichloromethane fraction of hydroalcoholic extract	Antifungal: *Paracoccidioides brasiliensis* (MFC: 30 µg mL^{-1})	biphenyl 4'-ethyl-4-methyl-2,2',6,6'-tetrahydroxy[1,1'-biphenyl]-4,4'-dicarboxylate	4
Fruits	Essential oil extraction by hydrodistillation	Antibacterial: *Corynebacterium* sp. (MIC: 3.55 µg mL^{-1}), *Bacillus* sp. (MIC: 7.11 µg mL^{-1}), *Nocardia* sp. (MIC: 7.11 µg mL^{-1}), *Enterobacter* sp. (MIC: 56.86 µg mL^{-1}), *Enterobacter agglomerans* (MIC: 28.43 µg mL^{-1}), *Escherichia coli* (MIC: 28.43 µg mL^{-1}), *Klebsiella oxytoca* (MIC: 28.43 µg mL^{-1})	monoterpenes (δ-3-carene, limonene, α-phellandrene, α-pinene) and Sesquiterpenes (trans-caryophellene)	5
Leaves	Dichloromethane extract	Antibacterial: *Staphylococcus aureus* (MIC: 600 µg mL^{-1}), *Pseudomonas aeruginosa* (MIC: 550 µg mL^{-1}), *Escherichia coli* (MIC: 850 µg mL^{-1}) Antifungal: *Aspergillus niger* (MIC: 750 µg mL^{-1}), *A. parasiticus* (MIC: 800 µg mL^{-1}), *Candida albicans* (MIC: 700 µg mL^{-1}),	monoterpenes and sesquiterpenes	6
	Methanol extract; hydroalcoholic solution	Antibacterial: *Streptococcus mutans* (MICA: 7 µg mL^{-1}) Antifungal: *Candida albicans* (MICA: 7 µg mL^{-1})	-	7
	Hexane fraction of hydroalcoholic extract	Antifungal: *Paracoccidioides brasiliensis* (MFC: 15-125 µg mL^{-1})	schinol	4

† SI: Selectivity Index; EC_{50}: 50% inhibitory concentration; MIC: minimum inhibitory concentration; MFC: minimum fungicidal concentration; MICA: Minimal inhibitory concentration of adherence (biofilm)
* "-": no information on the bioactive chemistry provided

Supplementary Table 3: 430D-F5 inhibits *agr* fluorescent reporter signaling with minimal growth (OD600nm) effects at concentrations < 64 μg mL^{-1}. Data are reported as percent vehicle control.

**Conc. 430D-F5 (μg mL^{-1})**	**OD600 % Vehicle Control (± Standard Deviation)**			
	***agr I* (AH1677)**	***agr II* (AH430)**	***agr III* (AH1747)**	***agr IV* (AH1872)**
**2**	110.4 ± 2.3	114.9 ± 2.4	104.7 ± 1.9	102.6 ± 0.7
**4**	114.5 ± 2.3	118.9 ± 4.0	111.5 ± 1.4	101.4 ± 1.9
**8**	120.5 ± 3.1	125.4 ± 6.1	115.6 ± 3.0	108.3 ± 1.4
**16**	120.0 ± 9.8	126.9 ± 5.4	111.8 ± 1.4	104.8 ± 3.8
**32**	121.6 ± 3.8	122.7 ± 1.4	100.2 ± 2.9	96.3 ± 7.6
**64**	92.5 ± 4.2	108.5 ± 1.7	69.5 ± 1.4	85.9 ± 3.6
**128**	65.8 ± 1.5	76.9 ± 4.9	56.5 ± 0.6	65.2 ± 1.6
**Conc. 430D-F5 (μg mL^{-1})**	**Fluorescence % Vehicle Control (± Standard Deviation)**			
	***agr I* (AH1677)**	***agr II* (AH430)**	***agr III* (AH1747)**	***agr IV* (AH1872)**
**2**	47.8 ± 4.2	51.5 ± 3.1	56.2 ± 1.7	83.5 ± 2.3
**4**	36.7 ± 2.3	33.3 ± 0.5	40.8 ± 0.8	64.7 ± 3.0
**8**	30.0 ± 1.1	25.1 ± 1.8	32.1 ± 0.5	54.9 ± 1.8
**16**	26.0 ± 4.6	18.6 ± 0.9	27.8 ± 0.3	51.4 ± 5.2
**32**	18.2 ± 2.2	14.6 ± 0.4	18.1 ± 0.3	41.3 ± 2.2
**64**	16.9 ± 0.8	14.3 ± 0.2	11.2 ± 0.3	27.0 ± 3.3
**128**	11.7 ± 0.3	13.3 ± 2.0	6.7 ± 0.2	21.0 ± 0.2

Supplementary Table 4: Negative and positive ESI Mass spectrometry (m/z) analysis of 430D-F5. The corresponding HPLC-DAD chromatogram is reported in Fig. 9a and putative structures in Fig. 9b.

Peak #	RT (min)	(%) Relative Abundance		Formula (Δ- ppm)[a]	Putative Compounds	ESI Mode	*m/z*[b]	MS[2]
		Neg. Mode	Pos. Mode					
**1**	39.78	0.00	0.55	$C_{54}H_{59}O_6$ (1.3)	no matches	+	391.2296, 413.2114, **803.4316**	ND
**2**	45.70	11.65	3.59	$C_{30}H_{17}O_{10}$ (0.2)	amentoflavone; agathisflavone; robustaflavone	**-**	537.0828	375.1374, 443.1003
**3**	46.28	8.39	2.14	$C_{30}H_{17}O_{10}$ (0.2)	see peak **1**	**-**	537.0826	375.1590, 443.1464
**4**	47.25	22.13	3.37	$C_{30}H_{21}O_{10}$ (1.4)	chamaejasmin; tetrahydroamentoflavone; tetrahydrorobustaflavone	**-**	**541.1147**, 1083.2380	389.1906, 415.1563
**5**	47.79	3.03	0.62	$C_{30}H_{21}O_{10}$ (-0.2)	see peak **3**	**-**	541.1138	389.1694, 415.1370
**6**	48.19	4.66	0.73	$C_{53}H_{95}O_{15}$ (-1.3)	no matches	**-**	485.3277, 531.3344, **971.6664**	941.8088, 951.2601, 971.7518
**7**	49.49	1.14	0	$C_{31}H_{45}O_7$ (-0.6)	no matches	**-**	529.3167	485.5292
**8**	51.12	0.53	0.93	$C_{45}H_{66}O_{15}$ (-0.3)	no matches	**+**	415.2112, 432.2376, 493.2250, **846.4404**	ND
**9**	51.43	0.27	0.54	$C_{29}H_{37}O_3$ (3.7)	no matches	**-**	**433.2764**, 455.2583	400.9561, 415.3339
**10**	52.01	3.5	0.43	$C_{30}H_{45}O_6$ (0.5)	no matches	**-**	501.3224	439.3959
**11**	53.10	0	0.31	$C_{48}H_{70}O_3$ (-9.6)	no matches	**+**	356.2793, **694.5253**	542.3829, 676.2984
**12**	53.53	0.98	1.31	$C_{40}H_{69}O_8$ (0.9)	no matches	**+**	356.2795, 377.2299, **677.4993**, 699.4816	557.3066, 647.4799
**13**	54.48	0	2.11	$C_{40}H_{69}O_8$ (2.5)	no matches	**+**	**677.5004**, 699.4831	525.4104, 5557.4151, 647.5023
**14**	55.01	5.33	2.33	$C_{30}H_{45}O_4$ (0.9)	albsapogenin; (3α,13α,14β, 17α,20S,24Z)-3-hydroxy-21-oxo-lanosta-8,24-dien-26-oic acid; (13α,14β,17α,20R,24Z)-3α-hydroxy-21-oxolanosta-8,24-dien-26-oic acid; (3α, 13α,14β,17α,24Z)-3-hydroxy-7-oxo-lanosta-8,24-dien-26-oic acid; mollinoic acid	**-**	**469.3327**, 515.3390, 939.6748	351.3897, 451.4088
**15**	55.44	2.14	2.33	$C_{30}H_{45}O_4$ (2.9)	see peak **13**	**-**	**469.3336**, 515.3379	ND
**16**	56.12	0.63	1.78	$C_{40}H_{61}O_6$ (0.9)	no matches	**-**	533.3491, **637.4479**, 683.4534	607.5950
**17**	56.58	7.99	8.52	$C_{30}H_{45}O_4$ (1.0)	see peak **13**	**-**	**469.3328**, 515.3391,	407.4528

Peak #	RT (min)	(%) Relative Abundance		Formula (Δ- ppm)[a]	Putative Compounds	ESI Mode	*m/z*[b]	MS^2
		Neg. Mode	Pos. Mode					
							939.6760	
**18**	57.76	1.52	2.86	$C_{30}H_{47}O_4$ (3.1)	see peak **13**	**+**	471.3483	453.4008
**19**	58.06	1.05	2.09	$C_{30}H_{45}O_4$ (3.4)	isomasticadienonalic acid	**+**	469.3328	260.1903, 332.2413, 452.3980
**20**	58.32	1.09	2.77	$C_{53}H_{97}O_{13}$ (2.1)	no matches	**+**	471.3485, 621.4551, **941.6944**	941.6543
**21**	58.48	2.07	0	$C_{30}H_{45}O_6$ (0.1)	no matches	**-**	501.322	439.3777
**22**	58.74	3.49	10.4	$C_{30}H_{45}O_4$ (2.2)	see peak **13**	**-**	**469.3333**, 515.3384, 939.6736	439.4702
**23**	59.35	2.39	3.43	$C_{30}H_{47}O_4$ (1.9)	see peak **13**	**+**	471.3488	217.1912, 435.3044, 453.3376
**24**	59.73	5.37	4.12	$C_{30}H_{45}O_5$ (2.1)	no matches	**-**	**485.3282**, 531.3338, 971.6649	453.3968, 467.3944
**25**	60.44	2.06	2.55	$C_{30}H_{45}O_4$ (0.1)	see peak **13**	**-**	**469.3323**, 515.3383, 939.6758	451.3724
**26**	60.75	1.23	1.48	$C_{30}H_{45}O_4$ (0.8)	see peak **13**	**-**	**469.3327**, 515.3375	451.3639
**27**	61.72	1.56	2.29	$C_{30}H_{47}O_5$ (1.5)	no matches	**-**	**487.3436**, 533.3478, 975.6950	455.4224, 469.4080

[a]The empirical formula corresponds to the $[M+H]^+$ or $[M-H]^-$ ion as determined by the ionization mode.
[b]The reported ions correspond to the $[M+H]^+$ or $[M-H]^-$ ion as determined by the ionization mode. When multiple ions were formed, the number in **bold font** indicates the $[M+H]^+$ or $[M-H]^-$ and underwent MS^2 analysis.

Supplementary Table 5: Description of bacterial strains used in the study.

Species	Strain ID	Characteristics†	Source*	Ref.
Corynebacterium amycolatum	SK46; HM-109	Isolated from the right arm normal skin of a 57-year-old male; a reference genome for the species. Genbank sequence: ABZU00000000	BEI; HMP	
Corynebacterium striatum	FS-1	Clinical isolate from Italy (2005-2007)	BEI	
Micrococcus luteus	SK58; HM-114	Isolated from normal skin of the left arm of a 57-year-old male; a reference genome for the species. Genbank sequence: ADCD00000000	BEI; HMP	
Propionibacterium acnes	HL005PA2; HM-493	Isolated from human skin; a reference genome for the species. Genbank sequence: ADZN00000000	BEI; HMP	
Staphylococcus aureus	LAC; AH1263	USA300		8
	UAMS-1; ATCC49230	Clinical isolate from osteomyelitis; USA200	MS	9
	UAMS-929	Δ*sarA* mutant of UAMS-1	MS	10
	AH1677	AH845 + pDB59 cm^R; *agr* type I YFP reporter; USA300	AH	11
	AH430	SA502a + pDB59 cm^R; *agr* type II YFP reporter;	AH	11
	AH1747	MW2 + pDB59 cm^R; *agr* type III YFP reporter	AH	11
	AH1872	MN EV (AH407) + pDB59 cm^R; *agr* type IV YFP reporter	AH	11
	AH2759	LAC + pAmiAgrP3 cm^R; *agr* P3-*lux* reporter	AH	12
	AH3052	Δ*spa* mutant of LAC	AH	13
	AH1589	Δ*hla* mutant of LAC	AH	14
	NRS225	Clinical isolate from UK (1976). *mecA*+; pen^R. CC121, ST121	NARSA	
	NRS230	Clinical isolate from 2 year old male in France (2002), associated with elbow arthritis with scarlet fever. *agr* type IV; *egc*+, (*lukE-lukD*)+, *hlgv*+, *mecA*-. erm^R, pen^R,. ST121-SLV, CC121	NARSA	
	NRS232	Clinical isolate (blood) from 4 year old male in Frnace (2002), associated with necrotizing pneumonia. *agr* type I; *egc*+, (*lukS-lukF PVL*)+, *hlg*+, *mecA*-; erm^I, gent^R, pen^R; 2 colony variants. ST22, CC22	NARSA	
	NRS242	Clinical isolate from 1 year old female in France (2002), associated with impetigo. *agr* type IV; *egc*+, (*lukS-lukF*)+ PVL+, (*lukE-lukD*+), *hlgv*+, *mecA*-. erm^I, pen^R. CC121, ST121	NARSA	
	NRS249	Clinical isolate (blood) from 5 year old male in France (2002), associated with native valve endocarditis. *agr* type I; *sea*+, *hlgv*+, *mecA*+. cipx^R, cli^R, erm^R, gen^R, oxa^R, pen^R. SCCmec type IV. ST247, CC8(247)	NARSA	
	NRS385; NR-46071	Clinical isolate from USA. USA500, *agr* type I, *sea*+, *seb*+, SCCmecIV, *mecA*+. cipx^R, cli^R, erm^R, gen^R, oxa^R, pen^R, tet^R, tmp/smx^R.	NARSA	15
Staphylococcus epidermidis	NIHLM001; HM896	Isolated in 2008 from an alar crease of a healthy 27-year-old white male volunteer in the United States; a reference genome for the species. Genbank sequence: AKHC01000000	BEI; HMP	

Species	Strain ID	Characteristics[†]	Source[*]	Ref.
	NRS101; RP62A; ATCC 35984	Clinical isolate from USA, catheter sepsis. Prototype biofilm producer, produces polysaccharide adhesin. *mecA*+; $clin^R$, erm^R, gen^R, oxa^R, pen^R	NARSA	
Staphylococcus haemolyticus	NRS116	Clinical isolate from 45 year old male in USA (2002); variant small colony type of NRS115; *mecA*+; van^I; tec^I	NARSA	
Staphylococcus warneri	SK66	Isolated from normal skin of right arm of healthy 57 year old male; a reference genome for the species. Genbank sequence: ACPZ00000000	BEI; HMP	
Streptococcus mitis	F0392	Isolated from human oral cavity; a reference genome for the species	BEI; HMP	
Streptococcus pyogenes	MGAS15252	Clinical isolate from thigh of patient with an invasive soft tissue infection in Canada (2008); serotypeM59, Group A *Streptococcus.* Genbank sequence: CP003116	BEI	

[†]**Characteristics:** CC: clonal complex; ST: Sequence Type; I: Intermediate; R: Resistant; cm: chloramphenicol; cipx: ciprofloxaxin; cli: clindamycin; erm: erythromycin; gen: gentamicin; oxa: oxacillin; pen: penicillin; tec: teicoplanin; tmp/smx: trimethroprim/sulfamethoxazole.van: vancomycin.

[*]**Source:** AH: A. Horswill, U. Iowa; BEI: Biodefense and Emerging Infections Research Resources Repository; HMP: Human Microbiome Project; NARSA: Network on Antimicrobial Resistance in *Staphylococcus aureus*; MS: M. Smeltzer, UAMS.

References Cited in Supplementary Files

1 Nocchi, S. R. *et al.* *In vitro* cytotoxicity and anti-herpes simplex virus Type 1 activity of hydroethanolic extract, fractions, and isolated compounds from stem bark of *Schinus terebinthifolius* Raddi. *Pharmacogn Mag* **12**, 160-164, doi:10.4103/0973-1296.177903 (2016).

2 Biasi-Garbin, R. P. *et al.* Antifungal potential of plant species from Brazilian Caatinga against dermatophytes. *Rev Inst Med Trop Sao Paulo* **58**, 18, doi:10.1590/S1678-9946201658018 (2016).

3 Moura-Costa, G. F. *et al.* Antimicrobial activity of plants used as medicinals on an indigenous reserve in Rio das Cobras, Parana, Brazil. *J Ethnopharmacol* **143**, 631-638, doi:10.1016/j.jep.2012.07.016 (2012).

4 Johann, S. *et al.* Antifungal activity of schinol and a new biphenyl compound isolated from *Schinus terebinthifolius* against the pathogenic fungus *Paracoccidioides brasiliensis*. *Ann Clin Microbiol Antimicrob* **9**, 1-6, doi:10.1186/1476-0711-9-30 (2010).

5 Cole, E. R. *et al.* Chemical composition of essential oil from ripe fruit of *Schinus terebinthifolius* Raddi and evaluation of its activity against wild strains of hospital origin. *Braz J Microbiol* **45**, 821-828 (2014).

6 El-Massry, K. F., El-Ghorab, A. H., Shaaban, H. A. & Shibamoto, T. Chemical compositions and antioxidant/antimicrobial activities of various samples prepared from *Schinus terebinthifolius* leaves cultivated in Egypt. *J Agric Food Chem* **57**, 5265-5270, doi:10.1021/jf900638c (2009).

7 Barbieri, D. S. V. *et al.* Antiadherent activity of *Schinus terebinthifolius* and *Croton urucurana* extracts on in vitro biofilm formation of *Candida albicans* and *Streptococcus mutans*. *Arch Oral Biol* **59**, 887-896, doi:10.1016/j.archoralbio.2014.05.006 (2014).

8 Boles, B., Thoendel, M., Roth, A. & Horswill, A. Identification of genes involved in polysaccharide-independent *Staphylococcus aureus* biofilm formation. *PLoS ONE* **5** (2010).

9 Gillaspy, A. *et al.* Role of the accessory gene regulator (*agr*) in pathogenesis of staphylococcal osteomyelitis. *Infect Immun* **63**, 3373-3380 (1995).

10 Beenken, K. E., Blevins, J. S. & Smeltzer, M. S. Mutation of *sarA* in *Staphylococcus aureus* limits biofilm formation. *Infect Immun* **71**, 4206-4211, doi:10.1128/IAI.71.7 (2003).

11 Kirchdoerfer, R. N. *et al.* Structural basis for ligand recognition and discrimination of a quorum-quenching antibody. *J Biol Chem* **286**, 17351-17358 (2011).

12 Figueroa, M. *et al.* Polyhydroxyanthraquinones as quorum sensing inhibitors from the guttates of *Penicillium restrictum* and their analysis by Desorption Electrospray Ionization Mass Spectrometry. *J Nat Prod* **77**, 1351-1358, doi:10.1021/np5000704 (2014).

13 Ibberson, C. B. *et al.* *Staphylococcus aureus* hyaluronidase is a CodY-regulated virulence factor. *Infect Immun* **82**, 4253-4264, doi:10.1128/iai.01710-14 (2014).

14 Olson, M. E. *et al.* *Staphylococcus aureus* nuclease is an SaeRS-dependent virulence factor. *Infect Immun* **81**, 1316-1324, doi:10.1128/iai.01242-12 (2013).

15 McDougal, L. K. *et al.* Pulsed-field gel electrophoresis typing of oxacillin-resistant *Staphylococcus aureus* isolates from the United States: Establishing a national database. *J Clin Microbiol* **41**, 5113-5120 (2003).
